# Nearly Aberration-Free Multiphoton Polymerization into Thick Photoresist Layers

**DOI:** 10.3390/mi8070219

**Published:** 2017-07-13

**Authors:** Bence Horváth, Pál Ormos, Lóránd Kelemen

**Affiliations:** Biological Research Centre, Institute of Biophysics, Hungarian Academy of Sciences, Temesvári krt. 62, 6726 Szeged, Hungary; horviratus@gmail.com (B.H.); ormos.pal@brc.mta.hu (P.O.)

**Keywords:** laser materials processing, hybrid manufacturing, two-photon polymerization, spatial light modulator, spherical aberration

## Abstract

In the era of lab-on-chip (LOC) devices, two-photon polymerization (TPP) is gaining more and more interest due to its capability of producing micrometer-sized 3D structures. With TPP, one may integrate functional structures into microfluidic systems by polymerizing them directly inside microchannels. When the feature of sub-micrometer size is a requirement, it is necessary to use high numerical aperture (NA) oil-immersion objectives that are optimized to work close to the glass substrate-photoresist interface. Further away from the substrate, that is, a few tens of micrometers into the photoresist, the focused beam undergoes focal spot elongation and focal position shift. These effects may eventually reduce the quality of the polymerized structures; therefore, it is desirable to eliminate them. We introduce a method that can highly improve the quality of structures polymerized tens of micrometers away from the substrate-photoresist interface by an oil-immersion, high NA objective. A spatial light-modulator is used to pre-compensate the phase-front distortion introduced by the interfacial refractive index jump on the strongly converging beam.

## 1. Introduction

**Figure 1:**
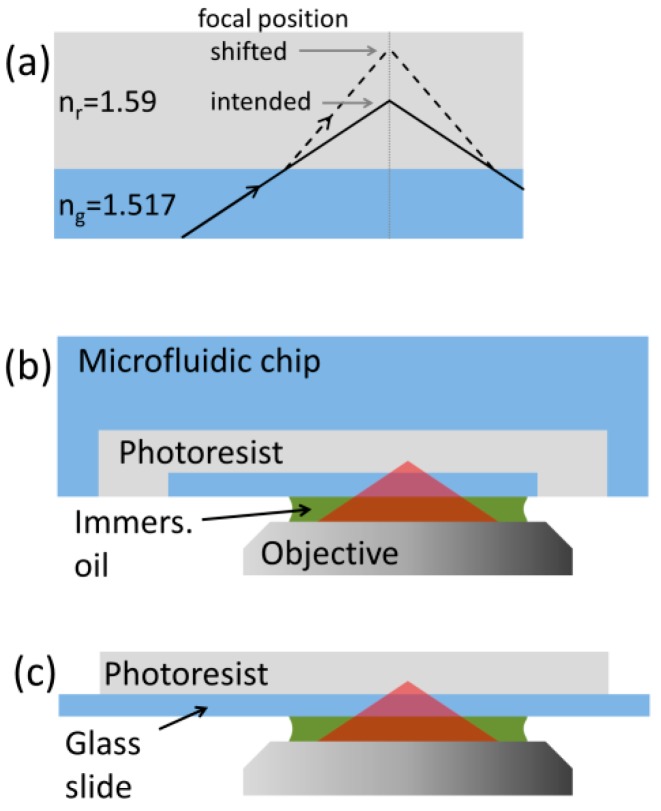
Focusing of a laser beam into a photoresist with a high NA objective. Panel (**a**) shows the marginal ray of the strongly focused beam passing through parallel, refractive-index mismatched layers. A possible scheme of TPP inside a microfluidic channel (**b**) and its model system on a microscope cover slide (**c**).

Lab-on-chip systems are important tools in various fields of basic research [1] and they are becoming key elements in several medical diagnostic devices [2]. Besides their intrinsic structural parts such as channels and reservoirs, their functionality can be greatly enhanced by specially designed microstructures embedded inside them. These functional elements can be passive (filters [3], lenses [4]) or active (pumps [5], valves [6], meters [7], or electrofluidic add-ons [8]). Since these elements cannot be delivered to the place where they perform their task, it is straightforward that they have to be prepared at their eventual position, inside the microchannels. One common way to create these functional parts is through laser-assisted polymerization out of a carefully chosen photoresist. Two-photon polymerization (TPP) is one of these methods recently used together with subtractive laser-based manufacturing techniques to create advanced, integrated LOC devices [9]. This method can provide complex 3D structures of typically a few tens of micrometers in size with a 100-nm feature size and arbitrary shape. The very small details of the structures made by TPP require the use of a high NA microscope objective to focus the polymerizing, usually a near infrared (NIR) laser beam with as small a diameter as possible. The most frequent high NA objectives used for TPP are those of oil immersion reaching NA 1.4. In these situations, however, the refractive index of the photoresist is usually higher (1.54–1.6) than that of the microchannel material (1.45 fused silica, ~1.52 glass), which causes focal spot distortion of the strongly converging beam travelling through these layers. The distortive effect is especially strong when the focal spot is positioned a few tens of micrometers into the photoresist. In this case, the focal spot shifts along the optical axis and distorts considerably, best illustrated by the large refraction of the marginal rays (Figure 1a).

This problem has already been reported in connection with various types of laser-assisted materials processing and even imaging. Nasse and Woehl discussed in detail the shape and distortions of the illumination point spread function (PSF) for scanning confocal microscopy when focusing into water through immersion oil and a glass substrate [10]. With numerical methods, they analyzed the effect of objective-to-substrate distance, the substrate refractive index, and of course, the NA on the focal intensity distribution and position, and found all of these substantial. The focusing of the beam of a femtosecond (fs) pulsed-laser by Sun and coworkers [11] into a silica layer showed significant focal spot distortion and shift, even when relatively low (0.4) NA objective was used. The effect was lessened by focusing a divergent beam with the objective, thereby introducing an initial spherical aberration (SA). Jesacher and Booth [12] processed diamond with 3D laser direct writing using a 1.25 NA objective and developed an adaptive aberration correction method to compensate for the refractive index mismatch at the immersion oil-diamond interface. Their optical system included a spatial light modulator (SLM), an active diffractive optical element that can modify the wavefront of the used laser beam. This way they introduced a pre-compensating spherical aberration that was modulated according to the distance of the focal spot and the interface, and improved the precision of the diamond fabrication deep inside the material. A similar aberration was compensated for in multiphoton polymerization when Williams and his co-workers investigated this effect on the height and width of suspended polymerized lines inside a thick layer of photoresist [13]. Their solution for the focal spot distortion was the numerical calculation-assisted adaptive change of the power of the polymerizing laser beam to maintain similar geometric features throughout a 40-μm thick layer of SU8 photoresist. Obata and co-workers applied another noticeable approach to polymerize tall structures into a thick photoresist layer [14]: they polymerized into a liquid-phase photoresist in between two glasses, the distance of which could be changed. The distance of the focal spot and that of one of the glasses was fixed. The TPP of structures was started on the second glass, and by distancing this second glass, very tall structures could be realized without changing the optical conditions for the focal spot. While this method resulted in nearly distortion-free tall structures, it required an adaptor to be fixed onto the objective and its use is limited only to liquid photoresists and cannot be used for closed microfluidic chambers.

In this paper, we show a method that potentially enables good quality two-photon polymerization into a photoresist that fills up closed microfluidic channels (Figure 1b). As a model system, we polymerize a series of suspended lines onto glass microscope slides of various thicknesses (Figure 1c) with a glass-to-line distance of up to 70 μm using a high NA oil immersion objective. The test lines are polymerized with an uncorrected laser beam and also with a beam that is pre-compensated for spherical aberration using an SLM. We measure the thickness and height values of the lines as the function of the distance for the uncorrected and corrected cases to evaluate the effect of distortion and the efficacy of the compensation. In a sense, our method is a combination and extension of the works of Jesacher and co-workers [12] and Williams and co-workers [13], because we use an SLM to introduce a pre-compensation for the spherical aberration on the wavefront of the collimated beam and we use it to improve the quality of photopolymerized structures. However, we extend their method by illuminating the photoresist not directly from the direction of the resist but through a glass substrate, which is more common in TPP systems. In order to test how the correction may work in microchannels with different wall thicknesses, we use glass substrates of three different thickness. We show that this correction can greatly reduce the focal shift as well as the structural distortion at various heights associated with TPP into a thick resist layer.

## 2. Materials and Methods

We used the polymerization system as introduced before [15] with slight modifications. Shortly, it uses a femtosecond laser source (λ = 785 nm, rep. rate: 100 MHz, τ = 100 fs, C-Fiber A780, Menlo System, Martinsried, Germany) and its expanded beam first hits the surface of the SLM (Pluto NIR, Holoeye Photonics AG, Berlin, Germany), with a 1/e^2^ diameter of ~10 mm. After the SLM, the light is focused into the photoresist layer by an oil immersion objective (Zeiss Achroplan, Carl Zeiss, Oberkochen, Germany) with 100× magnification and 1.25 NA. The refractive index of the immersion oil was ~1.51 at 785 nm. The sample is translated in the three spatial dimensions by a piezo stage (P-731.8L, Physik Instrumente, Karlsruhe, Germany, moving range: 100 × 100 μm). The photoresist layers were prepared simply by pipetting about 160 mg SU8-100 photoresist onto pre-cleaned cover slides of different thickness: 100 μm, 145 μm, and 170 μm; the glass thicknesses were confirmed by scanning electron microscopy (JSM-7100F/LV, JEOL Ltd., Tokyo, Japan). The thickness of the SU8 layer, following a 48-h long soft-bake at 95 °C, was measured to be 100–150 μm. After the illumination with the fs laser, the SU8 layers were heat-treated at 95 °C, developed by mrDev600 (Microresist GmBH, Berlin, Germany), and rinsed with ethanol. The heart of our method is a reflective-type SLM that consists of a reflective back surface with micrometer-sized electrodes forming a matrix, a transparent upper electrode plate, and a liquid crystal layer in between the two. SLMs can shift the phase of the reflected beam at each matrix point independently, giving rise to arbitrary diffractive patterns encoding the function of, for instance, a lens, a blazed grating, or any complex diffractive element [16]. They are essential parts of holographic optical tweezers and are often used in TPP systems as well [15,17,18].

In order to assess the degree of distortion and its correction, we prepared a series of test objects in the form of individual lines stretching between two supporting walls (Figure 3a). The geometrical features of this type of structure have already been proved to be very easy to analyze [13]. The lines are 10 μm long, their distance is designed to be 5 μm in the *z* direction and 2 μm in the *y* direction. This ensures that they can be observed from both directions without any hindrance and both their thickness and height can be measured precisely. The whole structure is 79 μm high. We used three polymerizing laser powers: 4 mW, 6 mW, and 8 mW, measured at the objective entrance pupil that corresponds to 138 GW/cm^2^, 206 GW/cm^2^, and 276 GW/cm^2^ intensity in the focus, respectively, considering 80% transmittance for the objective. The scan speed to illuminate the lines was chosen to be 40 μm/s as a medium speed that we normally use for our TPP experiments. According to a preliminary measurement, the tripled increase of the scan speed (from 20 μm/s to 60 μm/s) decreases line height by only about 25%, while only a double decrease of the power (from 8 mW to 4 mW) reduces it by a factor of nearly 2 (data not shown). Because of this more pronounced effect of laser power, we chose to vary only this process parameter in our study. According to recent findings, it is important to deal with the polarization direction of the polymerizing beam when focused by a high NA objective, since it has an effect on the feature size: it changes with the relative direction of the polarization and the scanning [19]. Rekštyte and co-workers found that a laterally scanned beam produces an approximately 10% broader line when the relative direction is perpendicular compared to the parallel case. Since our lines were straight, this direction did not change within one structure. The scanning direction was also kept constant between the experiments: it was always parallel to the polarization direction. Thereby, this effect did not interfere with our measurements. The observation of the structures was carried out with a scanning electron microscope (JSM-7100F/LV, JEOL Ltd., Tokyo, Japan).

**Figure 2:**
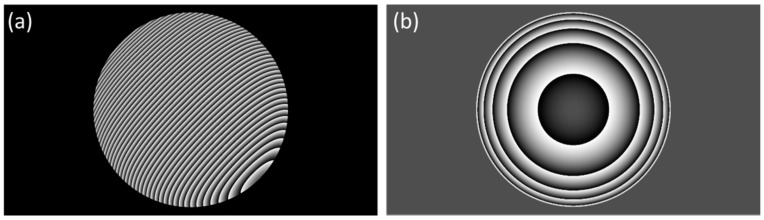
Phase shifting holograms for SA correction visualized as greyscale images. The hologram displayed on the surface of SLM (**a**) included a grating and a spherical aberration (**b**) part (here calculated for *z_m_* = 70 μm).

Since the distortion of the focal spot changes with its distance from the glass surface, we needed to apply various degrees of correction for the lines prepared at various heights. The correction was applied in the form of a phase shift pattern (Figure 2a) displayed on the SLM encoding a spherical aberration. The pattern, in addition to the SA (Figure 2b), also included a grating part and was calculated according to Jesacher et al. [12] in the following form:

(1) $${∆}_{m}\left(x,y\right)=\frac{2\pi }{\lambda_{0}f}\left(x{\hat{x}}_{m}+y{\hat{y}}_{m}\right)+{\hat{z}}_{m}SA\left(x,y\right)$$

where the first (grating) term shifts the focal spot to the ${\hat{x}}_{m},\;{\hat{y}}_{m}$ position in the sample plane (in our case, 10 μm along both the *x* and *y* axes relative to the position of the zero order), and the second term describes spherical aberration. The SA term, after being transformed into the pupil plane, has the following form:

(2) $$SA\left(r\right)=-\frac{2\pi }{\lambda_{0}f}\left[\sqrt{f^{2}n_{2}^{2}-r^{2}}-\sqrt{f^{2}n_{1}^{2}-r^{2}}\right]$$

In Equation (1), ${\hat{z}}_{m}$ is the position of the focal spot above the glass-photoresist interface (translated by the piezo scanner), *λ*_0_ is the wavelength of the polymerizing laser, *f* is the focal length of the objective, and *x* and *y* are the pixel coordinates on the SLM. In Equation (2), *n*_1_ and *n*_2_ are the refractive indices of the glass and the photoresist, respectively (*n*_1_ = 1.517, *n*_2_ = 1.59), and *r* is the radial coordinate in the pupil plane. A series of correction holograms were calculated in MATLAB (R2014b, The Mathworks Inc., Natick, MA, USA) for several values of ${\hat{z}}_{m}$, in 5-μm steps from 5 μm to 70 μm. The TPP process and the display of the actual correction hologram were controlled by a Labview program. The displaying Labview program monitored the actual *z* coordinate of the polymerized structure that was sent to the piezo stage and displayed the corresponding hologram.

## 3. Results

**Figure 3:**
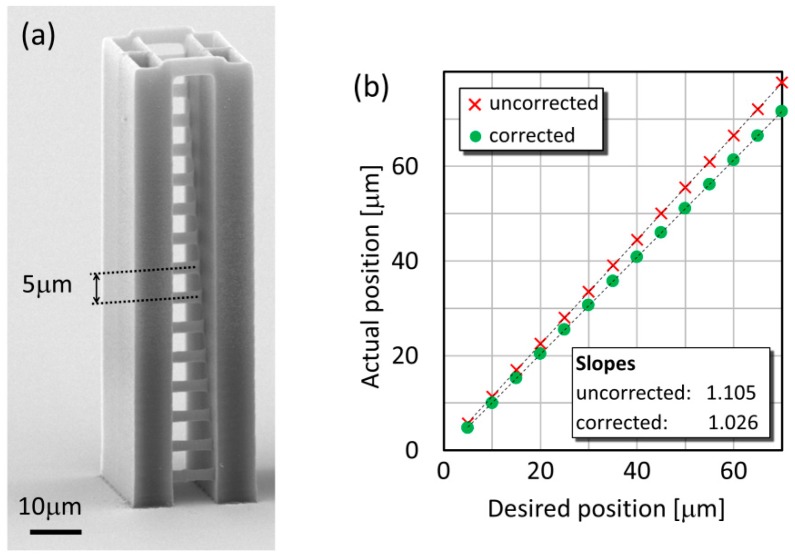
Test lines at various positions above the glass surface. Electron microscopic image of a test structure for the measurement of the geometrical features (**a**). Actual test line positions for the 170 μm glass with and without SA correction (**b**).

In our experiments, we used such power levels to prepare the hanging lines that produce continuous structures even at a distance of 80 μm from the substrate surface with the distorted beam. Therefore, the minimal applied power was 4 mW; in addition to this, we also used 6 mW and 8 mW. However, at 4 mW we observed that the lines are sometimes broken at the height of 80 μm, but at lower positions and at 6 mW or 8 mW for all positions, the lines are continuous. The eventual positions of the lines above the glass surface, determined with SEM, showed that the focal position is shifted upwards approximately by 6–10% for all applied parameters. The effect of focal shift is illustrated in Figure 3b, where the actual line positions prepared over a 170 μm glass are shown as the function of the desired positions. The points can be fitted with a linear curve, where the slopes demonstrate the magnitude of the focal shift. In this particular case, the focal shift for the uncorrected polymerization is 10.5%, which is reduced by about 8% with the SA correction. The average reduction was 7.75 ± 1.28% for this glass type, for 145 μm and 100 μm glasses it was 4.8 ± 0.82% and 7.14 ± 0.64%, respectively. A trivial calculation shows that, using a 1.25 NA objective in the system described in Figure 1a, the marginal rays focus about 14% higher that the intended focal position. The somewhat smaller shift observed in our measurements is probably due to the fact the marginal rays contribute less to the total polymerizing power considering an initial Gaussian beam profile. Overall, we can say that the SA correction always reduced the shift of the focal position and consequently that of the position of the polymerized structures.

**Figure 4:**
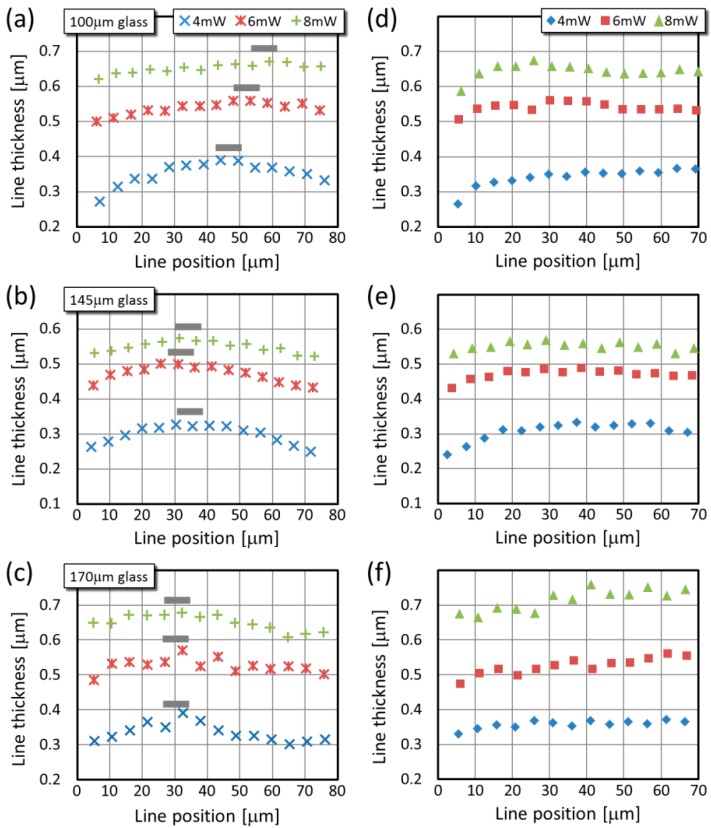
Typical line thickness values as the function of the position over the glass surface for three glass sizes. Panels (**a**–**c**) are from uncorrected lines, while panels (**d**–**f**) are from SA-corrected ones. The grey lines show the approximate positions of the maxima.

The geometrical features of the test lines made over the 100 μm, 145 μm, and 170 μm cover slides change with the distance above the glass surface. Regarding first the thickness of the hanging lines (Figure 4a–c), their values are greater when a larger laser power is used, as expected for the photoresist SU8: for 4 mW they are around 0.3 μm and for 8 mW they increase above 0.6 μm. The most prominent change in these values with the position over the glass surface is that they possess a maximum; this is true for all three types of glass and for all laser powers. The thickest lines (their positions are marked with grey lines in Figure 4a–c) are always situated between 30 μm and 55 μm above the glass surface, and their positions seem to shift slightly: on the 100 μm thick glass it is 45–55 μm above the glass, but on the 170 μm glass it is only about 30–35 μm above the glass. Interestingly, for the 100 μm glass this relatively large distance of the maximum position from the glass surface results in a line thickness drop between the maximum position and 80 μm that is relatively small. The variation of the line thickness is the most pronounced for 4 mW laser power on all types of glass: the thickness at the maximum is about 100 nm larger than at the minimum, which corresponds to 20–30% relative change. For the other two power levels, the highest variation is only 15% (145 μm glass, 6 mW power). These trends can be explained with the threshold behavior of the polymerization as follows. When the focal spot distorts upon the SA far from the glass surface (i) the intensity distribution broadens and (ii) the peak intensity drops relative to the ideal focusing conditions [13]. Closer to the glass surface, when the broadening and the intensity drop are small, that part of the focal spot where the intensity is still above the polymerization threshold becomes broader consequently the lines become broader. Further from the glass-SU8 interface, the intensity drop is dominant and it is above the polymerization threshold only at the very center of the focal spot, so the lines become narrow again. For the two larger power levels, after the intensity drop a wide part of the focal spot still remains above the threshold intensity even at 80 μm glass surface-focal spot distances; therefore, the line thickness is not reduced so much as in the 4 mW case.

The SA correction introduced a noticeable improvement in the variation of the line thickness described above. For all three glass types, the breakdown over the 30–55 μm position is practically removed and the curves flattened out, as illustrated in Figure 4d–f. This is the most visible for the lines made with 4 mW, but it also occurred at the other two power levels, albeit not so evidently since the original distortion was not so high. Interestingly, considering the 6 mW and 8 mW corrected data, in case of the thinnest type of glass (100 μm) there is a very small decrease above the 30 μm line position, for the 145 μm glass the curves are completely flat above this position, and for the 170 μm glass a very small increase is observed. In general, the line thickness between the 30 μm and 70 μm changes less than 5% after the correction. We have to note that the line thickness increase observed below 30 μm was not completely corrected with the method.

**Figure 5:**
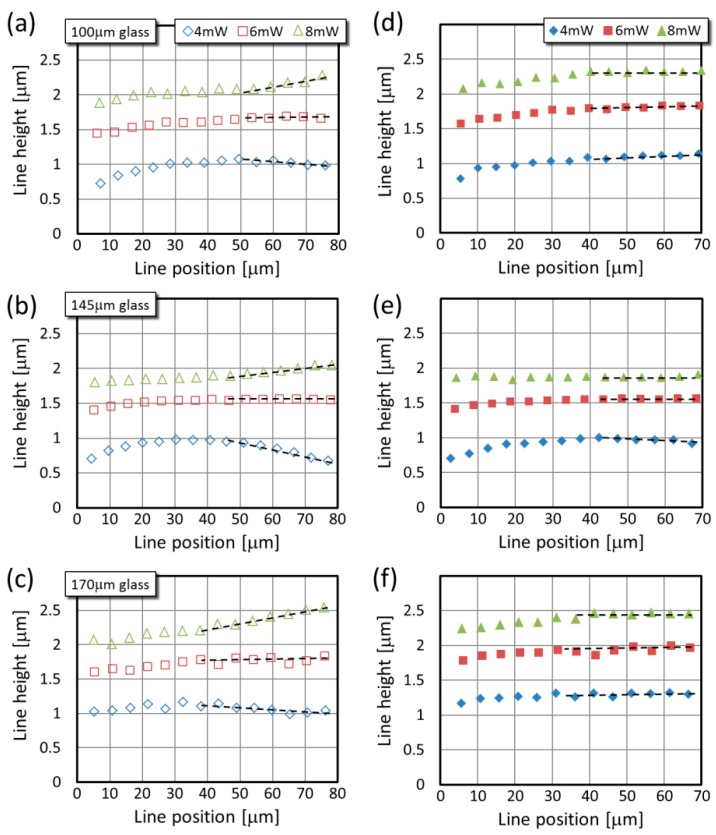
Typical line height values as the function of the position over the glass surface for three glass sizes. Panels (**a**–**c**) are from uncorrected lines, while panels (**d**–**f**) are from SA-corrected ones. The dashed lines are to guide the eye and to highlight the most prominent change upon the correction.

While the thickness of the uncorrected lines shows a similar trend with the glass surface-to-line distance for all three laser powers, their height changes in a different manner when different powers are applied (Figure 5a–c). Interestingly, when the line height values obtained at different glass thicknesses are compared, the tendency is similar at a given laser power. For all three types of glass, the data series of 4 mW power have maxima at around 30–50 μm above the glass surface, and it drops to 70–90% at larger distances. Although these maxima are less pronounced than those for the line thickness, it appears that their position shifts similarly as previously: from about 35 μm (4 mW) to about 50 μm (8 mW). In case of 6 mW laser power, the height values always level after an initial 7–10% increase. For each glass types, the onset of this plateau corresponds with the position of the maxima observed for the 4 mW data series. From the line height point of view, practically no correction is necessary for 6 mW laser power. The height of the lines made with 8 mW power increases continuously in the glass surface-to-line distance range. These main trends for the three powers are highlighted with dashed lines in Figure 5a–c.

The behavior of the line height can again be explained with the threshold behavior of the SU8 polymerization. The axial stretch of the focal intensity distribution at large glass surface-to-line distances reduces the intensity along the optical axis. For low laser powers, this means that the focal region where the intensity is larger than the polymerization threshold becomes shorter, while for large laser powers, the region with above-the-threshold intensity is still longer than in the aberration-free case. Therefore, for low powers at 80 μm distance the polymerized structures are shorter, while for large powers they are longer. We expect that for 6 mW and especially for 8 mW the intensity requires larger than 80 μm surface-to-line distance to achieve sufficient reduction distortion so that it falls below the threshold and the lines become shorter.

Upon the SA correction, the variation of the line height with the glass surface-line distance is practically eliminated for large distances: the increase for the 8 mW and the decrease for the 4 mW are flattened as shown again with the practically horizontal dashed lines in Figure 5d–f. Occasionally some minimal variation remains in the data, as exemplified with the small increase for the 4 mW data series between 45 μm and 70 μm in Figure 5d, but the general trend is observable for all parameters.

When the uncorrected and corrected graphs are compared in Figure 4 and Figure 5, one can observe that the thickness and height change of the lines close to the glass surface could be corrected only for a small extent. This change appears as an increase for all parameters for both the thickness and height. We speculate that this part is not due to the refractive index mismatch between the glass and the SU8 photoresist. According to the information form the manufacturer of the cover slide, the refractive index of the glass is 1.517 for 785 nm wavelength [20,21]; however, that of the immersion oil is 1.509. We speculate that this slight mismatch can cause the uncorrectable variance observed at lower positions.

## 4. Conclusions

In this paper, we introduced a method that can improve the quality of two-photon polymerized structures prepared in a photoresist layer over glass at various distances from the glass-resist interface. The method is based on the correction of the focal spot originally distorted by the refractive index mismatch between the glass and the resist. The distortion was described as a spherical aberration and was pre-compensated using a spatial light modulator to modify the phase front of the collimated polymerizing laser beam. The SLM added an inverted SA term to the phase-front that was adaptively modulated with the distance between the glass surface and the desired TPP structure. Compared to earlier results on the correction of the TPP method inside thick photoresist layers [13], our method optically corrects the focal spot distortion itself and the illumination is applied from the direction of the substrate. Our results on linear test structures show that the described correction method greatly reduced the distance-dependent variation of the line thickness and completely eliminated the height change far from the glass surface, while the procedure can still be improved close to the glass-resist interface. The application of this method on functional structures is currently under way.
